# Molecular survey and phylogenetic analysis of *Borrelia theileri*, *Rickettsia aeschlimannii*, *Mycoplasma ovis*, and *Mycoplasma wenyonii* in sheep and goats from southern Egypt

**DOI:** 10.1038/s41598-025-32438-4

**Published:** 2025-12-29

**Authors:** Hassan Y. A. H. Mahmoud, Ahmed M. Soliman, Wafa Abdullah I. Al-Megrin, Hind Alzaylaee, Tetsuya Tanaka, Walaa F. A. Emeish

**Affiliations:** 1 Division of Infectious Diseases, Animal Medicine Department, Faculty of Veterinary Medicine, Qena University, Qena, 83523 Egypt; 2 Biotechnology Department, Animal Health Research Institute, Agricultural Research Center, Dokki, Giza, 12618 Egypt; 3 Laboratory of Infectious Diseases, Joint Faculty of Veterinary Medicine, Kagoshima University, Kagoshima, 890-0065 Japan; 4 Department of Biology, College of Science, Princess Nourah bint Abdulrahman University, P.O. Box 84428, 11671 Riyadh, Saudi Arabia; 5 Laboratory of Animal Microbiology, Graduate School of Agricultural Science, Tohoku University, Sendai, 980-8572 Japan; 6 Medicine of Aquatic Life Department, Faculty of Veterinary Medicine, Qena University, Qena, 83523 Egypt

**Keywords:** Borrelia, Egypt, Goat, Mycoplasma, Rickettsia, Sheep, Infectious-disease diagnostics, Pathogens

## Abstract

Sheep and goats constitute a critical component of the Egyptian agricultural economy, serving as primary sources of milk, meat, and wool for rural populations. This study was conducted to detect and characterize infections caused by *Borrelia theileri*, *Rickettsia aeschlimannii*, *Mycoplasma ovis*, and *Mycoplasma wenyonii* in small ruminants from southern Egypt using molecular methods. The identification of these pathogens underscores potential threats to livestock productivity and raises public health concerns, particularly due to the zoonotic nature of *Rickettsia aeschlimannii*. The detection of these pathogens was performed using polymerase chain reaction assays targeting the *flaB, gltA*, and *16S rRNA* genes, enabling precise identification and genetic characterization of the infectious agents. Confirmation and genotyping were achieved through bidirectional sequencing and phylogenetic analysis. Blood samples were collected from 300 sheep and 300 goats across two governorates in southern Egypt. The overall infection rate of *Borrelia theileri* was 2.34% in sheep and 1.00% in goats. For *Rickettsia aeschlimannii*, the infection rates were 2.00% in sheep and 1.33% in goats. *Mycoplasma ovis* was detected in 1.33% of sheep and 1.00% of goats, while *Mycoplasma wenyonii* was present in 1.33% of sheep but not detected in goats. Genetic analysis indicated that the *Rickettsia aeschlimannii gltA gene and the Borrelia theileri flaB* genes were identical to those found in dogs, camels, and ticks in southern Egypt. The *Mycoplasma wenyonii 16S rRNA* gene matched cattle samples from the same region, and the *Mycoplasma ovis 16S rRNA* gene was identical to the one found in camels. Beyond confirming the presence of tick-borne diseases in small ruminants, these results show that tick-borne diseases are genetically related to other hosts present in the area. These findings highlight the need for integrated vector surveillance and host health assessments to evaluate zoonotic risk and subclinical impacts.

**Supplementary Information:**

The online version contains supplementary material available at 10.1038/s41598-025-32438-4.

## Introduction

Sheep and goats contribute approximately 25% of red meat and 14% of milk production in Egypt, highlighting their critical role in rural livelihoods. Small ruminants thrive in Egypt’s dry and semi-arid regions, making them indispensable for sustainable livestock production where large-scale cattle farming is unfeasible^1,2^. The health and production of small ruminants are seriously threatened by tick-borne diseases, especially in tropical and subtropical areas where tick vectors are endemic^3^. *Borrelia theileri*, transmitted by *Rhipicephalus* ticks, causes anemia and reduced productivity in small ruminants^4,5^. Infections can cause anemia, decreased productivity, and economic losses due to reduced reproduction, weight loss, and heightened vulnerability to secondary infections^4^. The hot climate and small ruminant farming in southern Egypt create favorable conditions for tick proliferation, increasing the risk of *Borrelia theileri* transmission^6^. The prevalence, genetic diversity, and impact of *Borrelia theileri* in sheep and goats, as well as its effects on small ruminants, remain understudied in Egypt^7^.

Arthropods, especially ticks, are the main transmission vectors for the obligate intracellular, Gram-negative, zoonotic bacteria that comprise the Spotted Fever Group^8^. *Rickettsia aeschlimannii* has become a disease of veterinary and public health concern. It was initially discovered in Moroccan *Hyalomma marginatum* ticks^9^. There is growing evidence that this bacterium causes diseases in humans and animals, and it has been found in ticks in Africa, Europe, and Asia^10^. Food security depends on the production of small ruminants, and as of right now, the epidemiology of *Rickettsia aeschlimannii* in small ruminants is not well understood^11^. The main vector of *Rickettsia aeschlimannii* is the *Hyalomma* tick species, which is supported by the arid climate of southern Egypt^12^. Although *Rickettsia aeschlimannii* has been identified in dog blood, ticks, and camel blood in earlier research in Egypt^13,14^, there is a dearth of information on its frequency in sheep and goats, even though these animals may serve as reservoirs of human infection. However, clinical signs of *Rickettsia aeschlimannii* infection in ruminants may include fever and decreased productivity^15,16^.

The erythrocyte-associated, epicellular bacteria known as hemotropic mycoplasmas (hemoplasmas) are a growing concern for human and animal health^17^. Hemotropic mycoplasmas, particularly *Mycoplasma ovis* are a significant concern globally due to their role in causing infectious hemolytic anemia in sheep and goats, which can result in substantial economic losses through increased mortality, decreased body weight, and lower yields of wool and meat^17^. Hemoplasma infections are often subclinical but may induce hemolytic anemia under immunosuppression or co-infection^18^. Several clinical manifestations of mycoplasmosis occur in livestock, including pneumonia, arthritis, and mastitis^19^. Several economic losses are associated with the disease, especially in cases of high morbidity and mortality^20^. *Mycoplasma ovis* is especially important in small ruminants because it can cause decreased milk production and reproductive losses^21^. Infection in ewes has been associated with higher lamb mortality, reduced weight gain, and abortion^22^. The environment and high tick infestation rates in southern Egypt make it a good condition for *Mycoplasma ovis* to spread^23^. Yet, there is still a lack of genetic epidemiological data, only one investigation has shown sequences of Mycoplasmas like ovis in Egyptian camels and ticks^24^. Furthermore, *Mycoplasma wenyonii* and similar species have been found in cattle and other livestock^25,26^, while candidatus *Mycoplasma haemolamae* was recently found in camels^27^. The clinical manifestations of *Mycoplasma wenyonii* in small ruminants remain poorly characterized to date. However, in large ruminants, the infection is known to present a variety of signs, including anemia, stunted growth, fever, lethargy, enlarged lymph nodes, depression, diarrhea, a noticeable drop in milk yield, infertility, swelling in the scrotum and hind limbs, and overall weight loss^28,29^.

This study molecularly investigates the infection rates, genetic diversity, and phylogenetic relationships of *Borrelia theileri*, *Rickettsia aeschlimannii*, *Mycoplasma ovis*, and *Mycoplasma wenyonii* in sheep and goats from southern Egypt to characterize molecular diversity and evolutionary relationships of sheep and goat pathogens, and evaluates their zoonotic potential through molecular analysis.

## Materials and methods

### Study design, research area, and collection of samples

**Fig. 1:**
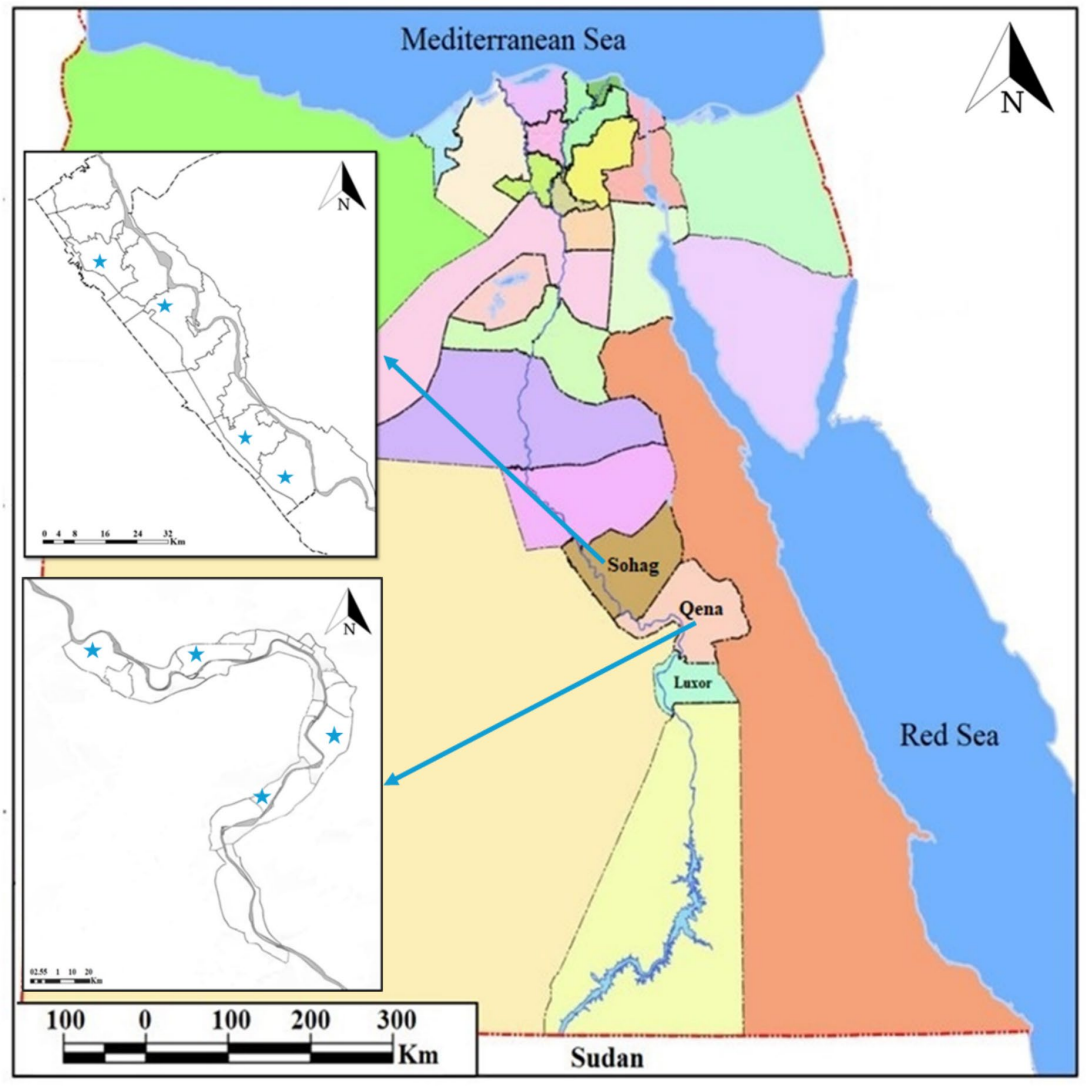
This map shows the areas in southern Egypt where samples were collected from two governorates, Sohag and Qena. Each region included four sampling sites. Blue stars indicate the locations where samples were collected in this study.

The Ethical Research Committee of South Valley University, Faculty of Veterinary Medicine, approved the experimental protocol and procedures that complied with applicable rules and regulations (Approval No. 10/09.02.2021). This study complied with the Applicable Animal Research Reporting of In Vivo Experiments (ARRIVE) requirements for animal research. A study was carried out between January 2022 and January 2023 to identify diseases in local sheep and goat breeds caused by vector-borne pathogens in southern Egypt in two governorates, Sohog and Qena (26° 33’ 32.6664″ N—31° 41’ 44.4156’' E, 26° 10’ 12″ N—32° 43’ 38″ E) **(**Fig. 1**)**. The sample size and sex distribution were primarily determined by owner cooperation and the accessibility of animals at the sampling sites, which ultimately defined the number of animals included, their population structure, and groupings. The 300 sheep of local breeds (100 females and 200 males) and 300 goats of local breeds (100 females and 200 males) ranged in age from six months to five years. The samples were obtained from small-scale owners with 10 or 20 animals. Using sterile, clean vacutainer tubes containing EDTA, whole blood samples for PCR amplification were drawn from the animal jugular vein. The samples were then kept at −20 °C until use. We chose the number of samples, populations, and groupings based on the cooperation of animal owners and accessibility.

### Clinical examination of animals

To sample collection, each animal underwent a thorough clinical assessment, which included determining age, examining mucous membranes, and measuring respiratory rate, pulse rate, and body temperature. These evaluations were conducted in accordance with standardized procedures for farm animal health checks, involving visual inspection, palpation, auscultation, and vital sign monitoring, as outlined by Jackson and Cockcroft^30^.

### Pathogen detection by PCR

First, we used a DNA extraction kit (Wizard® Genomic DNA Purification Kit, USA) to extract DNA from 600 blood samples. A Nanodrop 2000 spectrophotometer (Thermo Scientific, Waltham, Massachusetts, USA) was then used to measure the concentration of the extracted DNA. The Flagellin (*flaB*) gene of *Borrelia theileri*^31^, the citrate synthase (*gltA*) gene of *Rickettsia aeschlimannii*^32^, and the *16S rRNA* gene, which specifically targets *Mycoplasma ovis* and *Mycoplasma wenyonii*^33^, were then targeted by PCR using forward and reverse primers **(**Tables 1 and 2**).** For every PCR reaction, stringent contamination prevention measures were implemented by including negative controls containing nuclease-free water. Using 5 µl of 2 × Gflex PCR buffer, 0.5 µl of Tks Gflex DNA polymerase (TaKaRa), 0.5 µl of each forward and reverse primer with a concentration of 10 µM, 3 µl of nuclease-free water, and 0.5 µl of the template (DNA template with a concentration ranging from 10 to 30 ng/µl), the PCR reaction was conducted at a total volume of 10 µl. Negative samples in the form of nuclease-free water were employed as negative controls. PCR products were electrophoresed using an electrophoresis apparatus (Mupid Co., Ltd., Tokyo, Japan) in a 1.5% agarose gel in 1 × Tris–acetate-EDTA (TAE) buffer. Gel bands were visualized using a gel documentation system UV device, WUV-M20 (ATTO Co., Ltd., Tokyo, Japan), after staining with 5 µg/ml ethidium bromide in 1 × TAE.

**Table 1:** The primers used for the amplification of target fragments of genes in the present study.

Pathogen	Target gene	Sequence (5’−3’)	Product size (Pb)	References
***Borrelia theileri***	**Flagellin (*flaB*) gene**	GATCA(G/A)GC(T/A)CAA(C/T)ATAACCA(A/T)ATGCA AGATTCAAGTCTGTTTTGGAAAGC	344	(Takano et al., 2010)
		GCTGAAGAGCTTGGAATGCAACC TGATCAGTTATCATTCTAATAGCA		
***Rickettsia aeschlimannii***	**Citrate synthase (*gltA*) gene**	ATGACCAATGAAAATAATAAT CTTATACTCTCTATGTACA	341	(Mediannikov et al., 2004)
		GGGGGCCTGCTCACGGCGG ATTGCAAAAAGTACAGTGAAC		
**Mycoplasma species**	*16S rRNA*	ATACGGCCCATATTCCTACG TGCTCCACCACT TGT TCA	595–618	(Criado-Fornelio et al., 2003)

**Table 2:** PCR conditions for the amplification of target fragments of genes.

Pathogen	Target gene	Thermal Profile
*Borrelia theileri*	**Flagellin (*flaB*) gene**	(95 °C)/(5 min) → [(94 °C)/(30 s)-(55 °C)/(30 s)-(72 °C)/30 s] 40 × → (72 °C)/(5 min) → 10 °C → ∞ (95 °C)/(5 min) → [(94 °C)/(30 s)-(55 °C)/(30 s)-(72 °C)/30 s] 40 × → (72 °C)/(5 min) → 10 °C → ∞
*Rickettsia aeschlimannii*	**Citrate synthase (*gltA*) gene**	(95 °C)/(10 min) → [(94 °C)/(30 s)-(52 °C)/(30 s)-(72 °C)/90 s] 30 × → (72 °C)/(5 min) → 10 °C → ∞ (95 °C)/(5 min) → [(94 °C)/(30 s)-(52 °C)/(30 s)-(72 °C)/30 s] 30 × → (72 °C)/(5 min) → 10 °C → ∞
*Mycoplasma* species	***16S rRNA***	(95 °C)/(5 min) → [(95 °C)/(30 s)-(60 °C)/(30 s)-(72 °C)/30 s] 40 × → (72 °C)/(10 min) → 10 °C → ∞

### Sequence and data analysis

PCR reaction was done using 50 μl mixes to undertake sequence analysis to detect the *Borrelia theileri flaB* gene, the Rickettsia species *gltA* gene, and the hemotropic mycoplasma *16S rRNA* gene*.* Amplified gene fragments were extracted from agarose gels using the NucleoSpin Gel and PCR Clean-up kit (Macherey–Nagel, Duren, Germany) according to the manufacturer’s instructions. DNA concentrations were measured with a Nanodrop 2000 spectrophotometer (Thermo Fisher Scientific, Waltham, MA, USA). The purified DNA was submitted to Eurofins NSC Japan KK (Kanagawa, Japan) for bi-directional sequencing using the 3730 × 1 DNA Analyzer (Thermo Fisher Scientific). The resulting sequences were processed using SnapGene Viewer (GSL Biotech, Boston, USA) and MEGA X. Forward and reverse reads were aligned, merged, and compared to GenBank reference sequences via BLAST. Multiple sequence alignments were conducted using ClustalW in MEGA X, followed by trimming and model selection to determine optimal evolutionary models. Phylogenetic trees were generated using the maximum likelihood method with 1000 replicates to assess the robustness of the tree topology^34,35^. Statistical analysis was conducted, and 95% confidence intervals were calculated using www.vassarstats.net, the Fisher’s exact test was used for *Mycoplasma ovis* and *Mycoplasma wenyonii* due to low expected numbers, the Chi-square test was used to compare the infection rates and assess the association between categorical variables. A p-value of less than 0.05 was considered statistically significant^36^.

## Results

This study was conducted to molecularly detect the infection rates of *Borrelia theileri, Rickettsia aeschlimannii, Mycoplasma ovis*, *and Mycoplasma wenyonii* in blood samples collected from sheep and goats in southern Egypt. In sheep, 21 of 300 animals were infected with at least one pathogen, yielding an overall infection rate of 7.0%. In contrast, only 10 out of 300 goats were infected, resulting in a lower infection rate in goats than in sheep (3.33%) (p = 0.032). *Borrelia theileri* infection rates were 2.34% (7/300) in sheep and 1.00% (3/300) in goats, but this difference was not statistically significant (p = 0.332). *Rickettsia aeschlimannii* was detected in 6 sheep (2.00%) and 4 goats (1.33%) with no significant difference between the species (p = 0.751). In comparison, *Mycoplasma ovis* was detected in 4 sheep (1.33%) and 3 goats (1.00%), with no significant association (p = 1.000). However, *Mycoplasma wenyonii* was detected in 4 sheep (1.33%) but was undetected in goats (0.00%), a statistically significant difference was observed for *Mycoplasma wenyonii* (p = 0.045). Furthermore, there was no significant difference in infection rates between Sohag (5.33%, 16/300) and Qena (5.00%, 15/300) (p = 0.855) across geographical locations **(**Table 3**).** An analysis of infection risk factors across animal species and geographic regions revealed that species type had a statistically significant impact on infection rates, whereas location did not. Sheep exhibited an overall infection rate of 7.00%, more than twice that of goats at 3.33%, with a relative risk of 2.10 (p = 0.032). Although sheep consistently showed higher susceptibility to *Borrelia theileri* (risk factors = 2.34), *Rickettsia asechlimannii* (risk factors = 1.50), and *Mycoplasma ovis* (risk factors = 1.33), these differences did not reach statistical significance. In contrast, *Mycoplasma wenyonii* was detected exclusively in sheep (1.33%, p = 0.045), indicating a significant species-specific predisposition. Geographic location did not significantly influence infection rates; Sohag and Qena reported similar prevalence levels (5.33% and 5.00%), with a relative risk of 1.07 (p = 0.855), suggesting comparable exposure risks between the two governorates.

**Table 3:** Positivity rates of the detected *Borrelia theileri, Rickettsia aeschlimannii, Mycoplasma ovis* and *Mycoplasma wenyonii* in the present study.

Organism	Animals	Infected/examined animals	Infection rate	P-Value	95% C I	Risk factor animal species	Risk factor
							**Location**
						**Sheep versus Goats**	**Sohag versus Qena**
***Borrelia theileri***	Sheep	7/300	2.34%	0.332	0.9%—4.8%	2.34	1.00
	Goats	3/300	1.00%		0.2%—2.9%		
***Rickettsia aeschlimannii***	Sheep	6/300	2.00%	0.751	0.7%—4.3%	1.50	1.00
	Goats	4/300	1.33%		0.4%—3.4%		
***Mycoplasma ovis***	Sheep	4/300	1.33%	1.000	0.4%—3.4%	1.33	1.30
	Goats	3/300	1.00%		0.2%—2.9%		
***Mycoplasma wenyonii***	Sheep	4/300	1.33%	0.045*	0.4%—3.4%	∞ (Infinite)	1.00
	Goats	0/300	0.00%		0.0%—1.2%		
**Total infection**	Sheep	21/300	7.00%	0.032*	4.2%—10.5%	2.10	-
	Goats	10/300	3.33%		1.6%—6.0%		
**Total by location**	Sohag	16/300	5.33%	0.855	3.1%—8.5%	-	1.07
	Qena	15/300	5.00%		2.8%—8.1%		

*P-Value Significance: A p-value < 0.05 indicates a statistically significant difference.

*Fisher’s Exact Test was used for *Mycoplasma ovis* and *Mycoplasma wenyonii* due to low expected counts; the standard Chi-square test was used for others.

The analysis for locations is based on the total number of infections (Sohag = 16, Qena = 15), assuming an equal sample size of 300 per location.

The p-value tests if the overall infection rate differs between Sohag and Qena.

The risk is calculated as infinite (∞) because no infections were found in goats (0%), making the relative risk calculation (Sheep %/Goats %) undefined and effectively infinite. This highlights an exclusive risk for sheep.

**Fig. 2:**
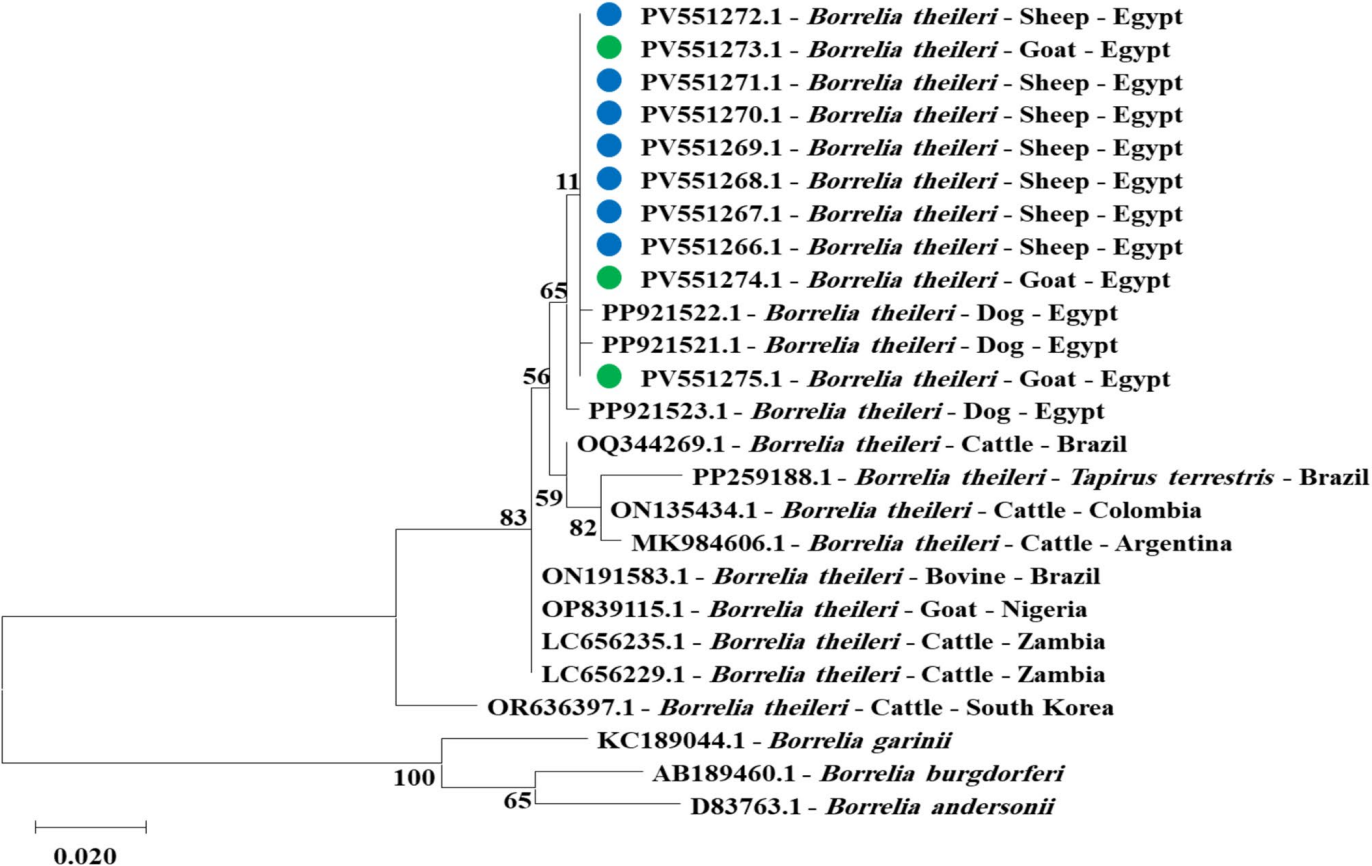
Phylogenetic relations of *Borrelia theileri* in sheep and goat obtained via the maximum-likelihood method and the Kimura three-parameter model based on flagellin (*flaB*) gene sequences. The percentage of trees on which the related taxa clustered is displayed next to the branches. Branch lengths are expressed in the number of substitutions per site, and the tree is drawn to scale. The blue circles represent *Borrelia theileri* in sheep, and the green circles represent *Borrelia theileri* in goats and the present study.

**Fig. 3:**
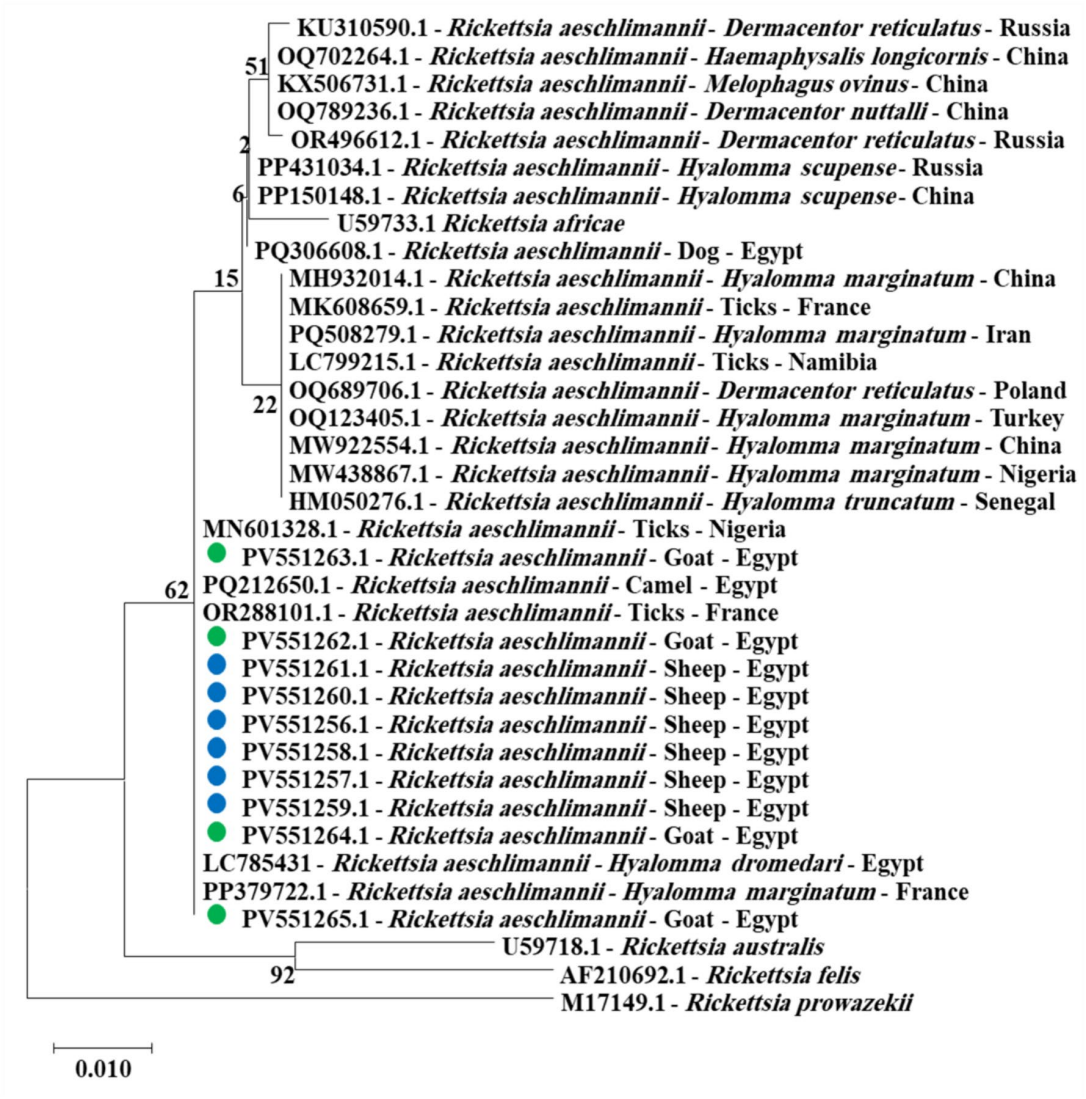
Phylogenetic relations of *Rickettsia aeschlimannii* in sheep and goat obtained via the maximum-likelihood method and the Kimura three-parameter model based on citrate synthase (*gltA*) gene sequences. The percentage of trees on which the related taxa are clustered is displayed next to the branches. Branch lengths are expressed in the number of substitutions per site, and the tree is drawn to scale. The blue circles represent *Rickettsia aeschlimannii* in sheep, and the green circle represents *Rickettsia aeschlimannii* in goats in the present study.

GenBank accession numbers from PV551266 to PV551275 contain the partial *flaB* gene sequences of *Borrelia theileri* in ten blood samples from sheep and goats. Sequences for these samples were identical at 100% and clustered closely with reference samples from dogs in the same region **(**Fig. 2** and **Fig. suppl. 1 and 2**)**. Furthermore, *Rickettsia aeschlimannii* partial *gltA* gene sequences from PV551256 to PV551265 for ten blood samples from sheep and goats showed 100% identity with previously reported samples from camels and ticks in southern Egypt **(**Fig. 3** and **Fig. suppl. 3**).**

**Fig. 4:**
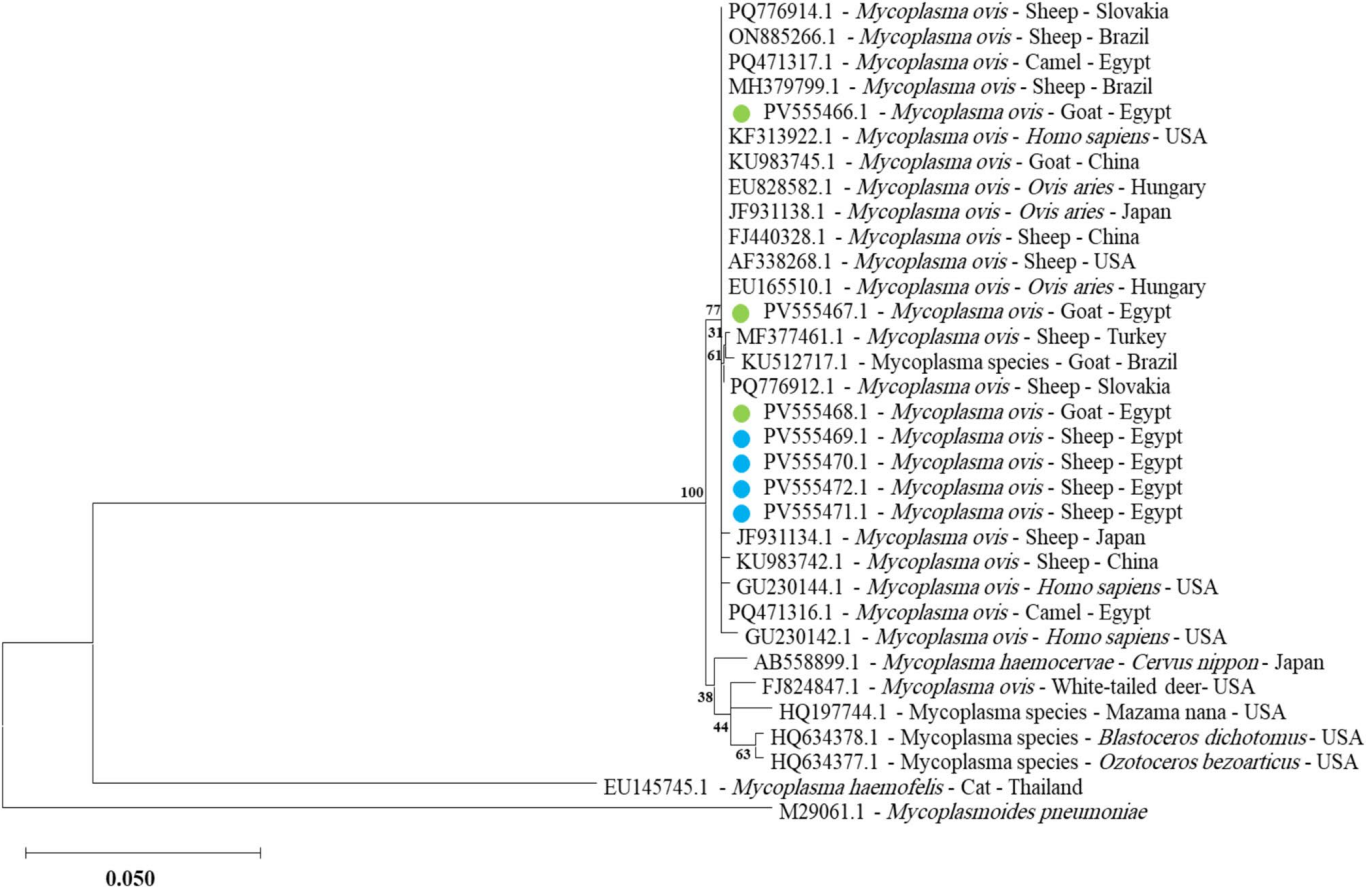
Phylogenetic relations of *Mycoplasma ovis* in sheep and goat obtained via the maximum-likelihood method and the Kimura three-parameter model based on *16S rRNA* gene sequences. The percentage of trees on which the related taxa are clustered is displayed next to the branches. Branch lengths are expressed in the number of substitutions per site, and the tree is drawn to scale. The blue circles represent *Mycoplasma ovis* in sheep, and the green circle represents *Mycoplasma ovis* in goats in the present study.

**Fig. 5:**
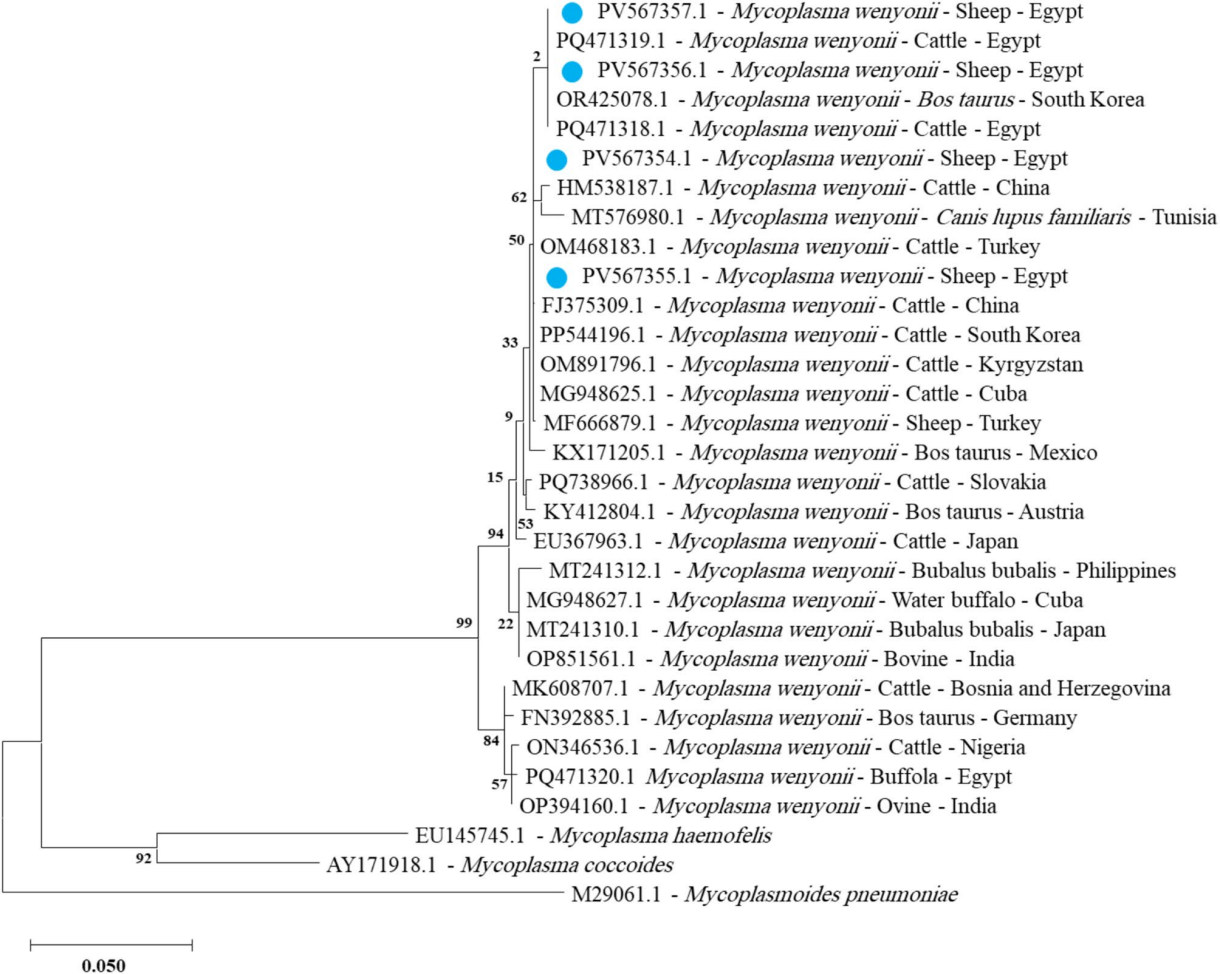
Phylogenetic relations of *Mycoplasma wenyonii* in sheep obtained via the maximum-likelihood method and the Kimura three-parameter model based on *16S rRNA* gene sequences. The percentage of trees on which the related taxa are clustered is displayed next to the branches. Branch lengths are expressed in the number of substitutions per site, and the tree is drawn to scale. The blue circles represent *Mycoplasma wenyonii* in sheep in the present study.

*Mycoplasma ovis* was detected in sheep and goats in seven blood samples with accession numbers from PV555466 to PV555472. These sequences, representing partial segments of the *16S rRNA* gene, showed 100% similarity to camel blood samples isolated in Egypt **(**Fig. 4** and **Fig. suppl.4**).** *Mycoplasma wenyonii* was also exclusively identified in sheep only in four blood samples with accession numbers from PV567354 to PV567357. Sequences of these samples were 99.59% to 100% identical to reference strains in GenBank and 100% identical to isolates from blood samples from cattle in Egypt **(**Fig. 5** and **Fig. suppl. 4**).**

## Discussion

Different infection rates of *Borrelia theileri, Rickettsia aeschlimannii, Mycoplasma ovis, and Mycoplasma wenyonii* were found in sheep and goats in the current investigation. According to this study, sheep had a higher overall infection rate (7.00%) than goats (3.33%), suggesting that host susceptibility, vector exposure, or environmental factors may affect the spread of pathogens differently across species. Sheep with higher infection rates may have physiological or behavioral characteristics that increase tick attachment, such as immunological responses, feeding habits, or wool density^37^. Conversely, goats may be more resistant to tick-borne pathogens, possibly due to grooming behaviors that reduce tick infestation^38,39^.

The prevalence of *Borrelia theileri* was lower in sheep (2.34%) and goats (1.00%) than in a previous study in dogs (5%) in the same region of southern Egypt^13^. The higher infection rate in sheep than in goats may reflect species-specific immune responses or increased tick infestation^40^. The higher infection rate has also been reported in cattle, where grazing animals exhibited higher *Borrelia theileri* infection rates than non-grazing animals^41,42^.

*Rickettsia aeschlimannii* was detected in (2.00%) of sheep and (1.33%) of goats, a rate lower than that found in the earlier study in Egypt in camels (5%) but higher than in dogs (1%) in southern Egypt^13^. *Rickettsia aeschlimannii* has also been identified in *Hyalomma dromedarii* (6.06%) in the same region^14^, and *Rickettsia aeschlimannii* was detected in various tick species infesting livestock in Northern Iran^43^. The broad host range of *Rickettsia aeschlimannii* raises zoonotic concerns, as it has been implicated in human infections^44^.

*Mycoplasma ovis* was detected in this study at lower rates in sheep (1.33%) and goats (1.00%) compared to camels (2.9%) in Egypt^24^ and goats (18.0%) in the United States^45^ and (4.5%) in Australia^46^, and 11.3% in Turkey^47^, and up to 100% in Mexico^48^. The lower infection rate in local breeds may indicate genetic resistance in these breeds to the infection.

*Mycoplasma wenyonii* was detected only in sheep (1.33%) and was not detected in goats. This result contrasted with higher prevalence rates detected in cattle (13.2%) and buffalo (4%) in southern Egypt^24^ and in other regions, such as Japan (21.8%^49^) and China (31%^50^). While hemotropic mycoplasmas are well documented in cattle, their occurrence in small ruminants remains less well characterized^51^. Further studies are needed to elucidate whether goats exhibit lower susceptibility or if these findings reflect epidemiological variability. The regions exhibit comparable climatic profiles, particularly in temperature ranges, which are critical determinants of pathogen survival, vector activity, and host susceptibility^52^. In some regions, similar practices in intensive farming, housing, grazing management, or biosecurity protocols can significantly reduce location-dependent risk factors, masking potential geographic variations^53^.

There is a significantly higher overall infection rate in sheep (7.00%) compared to goats (3.33%). This suggests an inherent greater susceptibility of sheep to these pathogens, potentially related to grazing behavior, immune response, or management practices. The species-specific risk is *Mycoplasma wenyonii*, which was observed only in sheep. This finding agrees with some previous studies that report a higher prevalence of *Mycoplasma wenyonii* in cattle and sheep compared to other ruminants^54,55^. The absence of *Mycoplasma wenyonii* detection in goats needs further investigation to determine whether this reflects true resistance or a lack of exposure to specific vectors.

For *Borrelia theileri, Rickettsia asechlimannii, and Mycoplasma ovis,* the infection rates were not statistically different between sheep and goats, although the calculated risk factor indicated a higher risk in sheep. The non-significant p-values for these pathogens are likely due to the low overall sample size, which reduces the statistical power to detect a significant difference.

The geographic locations of Sohag and Qena were not significant risk factors for the overall infection rate, suggesting that the ecological and environmental conditions favoring tick vectors and the transmission cycles of these pathogens are relatively homogeneous across the two governorates. Both geographic locations share similar climatic conditions and agricultural practices, which likely support comparable populations of tick vectors, such as *Rhipicephalus* and *Hyalomma* species, known to transmit these pathogens^56,57^.

The detection of *Borrelia theileri, Rickettsia asechlimannii, Mycoplasma ovis,* and *Mycoplasma* wenyonii in apparently healthy sheep and goats is a key finding of this study, indicating widespread subclinical infection. This state of endemic stability suggests that these animals act as reservoir hosts, maintaining the pathogen cycle in the region without displaying obvious illness^58^. However, subclinical infections can have substantial economic consequences due to insidious production losses and pose a risk of clinical manifestation under stressful conditions.

Genetic analysis of *Borrelia theileri* and *Rickettsia aeschlimannii* in this study provides valuable insights into the molecular epidemiology of these pathogens in southern Egypt. The partial *flaB* gene sequences of *Borrelia theileri* (GenBank accession nos. PV551266–PV551275) exhibited 100% identity among the sampled isolates and clustered closely with reference isolates deposited in GenBank from dogs in the same region (PP921521- PP921523). The high specificity and conservation across strains of *Borrelia* species led us to use the *flaB* gene in molecular diagnostics to detect and identify *Borrelia* species^59^. This high genetic similarity suggests an endemic circulation of *Borrelia theileri* among multiple animal species, including sheep, goats, and dogs, in southern Egypt. This result suggests that *Borrelia theileri* is maintained in a multi-host transmission cycle involving domestic animals and vectors^60^. The strong genetic identity of the *Borrelia theileri* samples isolated from sheep, goats, and dogs in southern Egypt suggests that little host-dependent genetic variation is an important finding of this study. This implies that dogs and small ruminants may be participating in a common transmission cycle, with dogs serving as reservoir hosts or aiding in the spread of *Borrelia theileri*. Furthermore, the identification of genetically identical strains of *Borrelia theileri* in several geographical locations supports the pathogen circulating as a common and adaptable infection that may infect a variety of mammalian hosts. These results highlight the crucial role of cross-species integrated surveillance in understanding the epidemiology of *Borrelia theileri* infections.

The partial *gltA* sequences of *Rickettsia aeschlimannii* (PV551256-PV551265) showed 100% identity and were found in the same cluster as previously reported and deposited in GenBank under MN601328.1 for ticks from Nigeria, LC785431 and PQ212650.1 for *Hyalomma dromedary* and camels from Egypt^13,14^. This genetic consistency indicates that *Rickettsia aeschlimannii* is widely distributed among hosts, including small ruminants, camels, and ticks, supporting its endemic persistence in the region. The detection of identical sequences across multiple host species suggests that species play a crucial role in the transmission of *Rickettsia aeschlimannii*^61^. The phylogenetic study clearly identifies the *Rickettsia aeschlimannii* found in Egyptian sheep and goats. A key observation is the absence of genetic differentiation linked to the host species. This strongly indicates that *Rickettsia aeschlimannii* functions as a generalist pathogen, sustained through a transmission cycle involving multiple tick species that feed on a wide array of mammalian hosts, including ruminants.

The molecular detection of *Mycoplasma ovis* and *Mycoplasma wenyonii* in small ruminants provides essential insights into the epidemiology of these hemotropic pathogens in Egypt. Our findings demonstrate that *Mycoplasma ovis* circulates in both sheep and goats, with partial *16S rRNA* gene sequences (GenBank accession nos. PV555466-PV555475) showing 100% identity to camel-derived isolates from Egypt. This high genetic identity strongly suggests interspecies transmission of *Mycoplasma ovis* among different livestock species in the region, possibly mediated by common vectors such as ticks or biting flies^22^. The detection of *Mycoplasma ovis* in multiple host species aligns with previous reports demonstrating its broad host range^62^. This finding underscores the need to continuously monitor *Mycoplasma ovis* infections in various animal species to better understand their transmission dynamics and potential impact on animal health.

*Mycoplasma wenyonii* was detected only in sheep (GenBank accession nos. PV567354-PV567357). The sequence of *Mycoplasma wenyonii* was identical to 100% with cattle isolates from Egypt (PQ471318 and PQ471319) and identical to 97.59%−100% with reference strains in GenBank. This finding suggests that either there are common reservoir hosts or environmental sources of infection, or that there is cross-species transmission due to vectors or management methods shared between cattle and sheep^62^. The absence of *Mycoplasma wenyonii* in the goats in the present study could be a sign of biological resistance or a limitation of the sample that warrants further research. This finding has significant implications for disease control, as it suggests that control strategies should target multiple host species simultaneously to effectively reduce transmission.

This study has some limitations, it only shows how common these infections are at one point in time. It cannot explain how the infections develop or change over time. Also, the study examined only a few specific pathogens and did not assess co-infections, which are often present in animals with tick-borne diseases. Even with these limits, the results are important. The higher risk in sheep underscores the need for better monitoring and tick control, especially in sheep flocks, to prevent problems such as anemia and reduced productivity. Veterinarians and farmers in the area should know that these pathogens are common and that *Mycoplasma wenyonii* seems to affect only sheep. Future studies should determine which vector types are spreading these infections in southern Egypt and how these pathogens affect animal health.

## Conclusion

A molecular detection study was conducted in southern Egypt for the first time in sheep and goats for *Borrelia theileri, Rickettsia aeschlimannii, Mycoplasma ovis,* and *Mycoplasma wenyonii.* Study results suggest that sheep are more susceptible to infections, which may lead to subclinical anemia and decreased productivity. Furthermore, interspecies transmission may pose a zoonotic risk. According to the molecular findings, these pathogens had strong genetic similarity across host species, indicating shared transmission cycles that arthropod vectors may facilitate. These findings demonstrate that, to manage these diseases effectively in Egyptian animal herds, combined management strategies targeting multiple host species and their vectors are required.

## Supplementary Information

Supplementary Information.

## Data Availability

Sequence data that support the findings of this study have been deposited in GenBank with the accession numbers PV555466-PV555472, PV551256-PV551275, and PV567354-PV567357.
