# Supplementary material for: Molecular survey and phylogenetic analysis of Borrelia theileri, Rickettsia aeschlimannii, Mycoplasma ovis, and Mycoplasma wenyonii in sheep and goats from southern Egypt
Source: Sci Rep. 2025 Dec 29;15:45665. doi: 10.1038/s41598-025-32438-4 (PMC12753633; doi:10.1038/s41598-025-32438-4)
Supplement: Supplementary file 1 — Supplementary Information. [file 41598_2025_32438_MOESM1_ESM.pptx]

## Slide 1
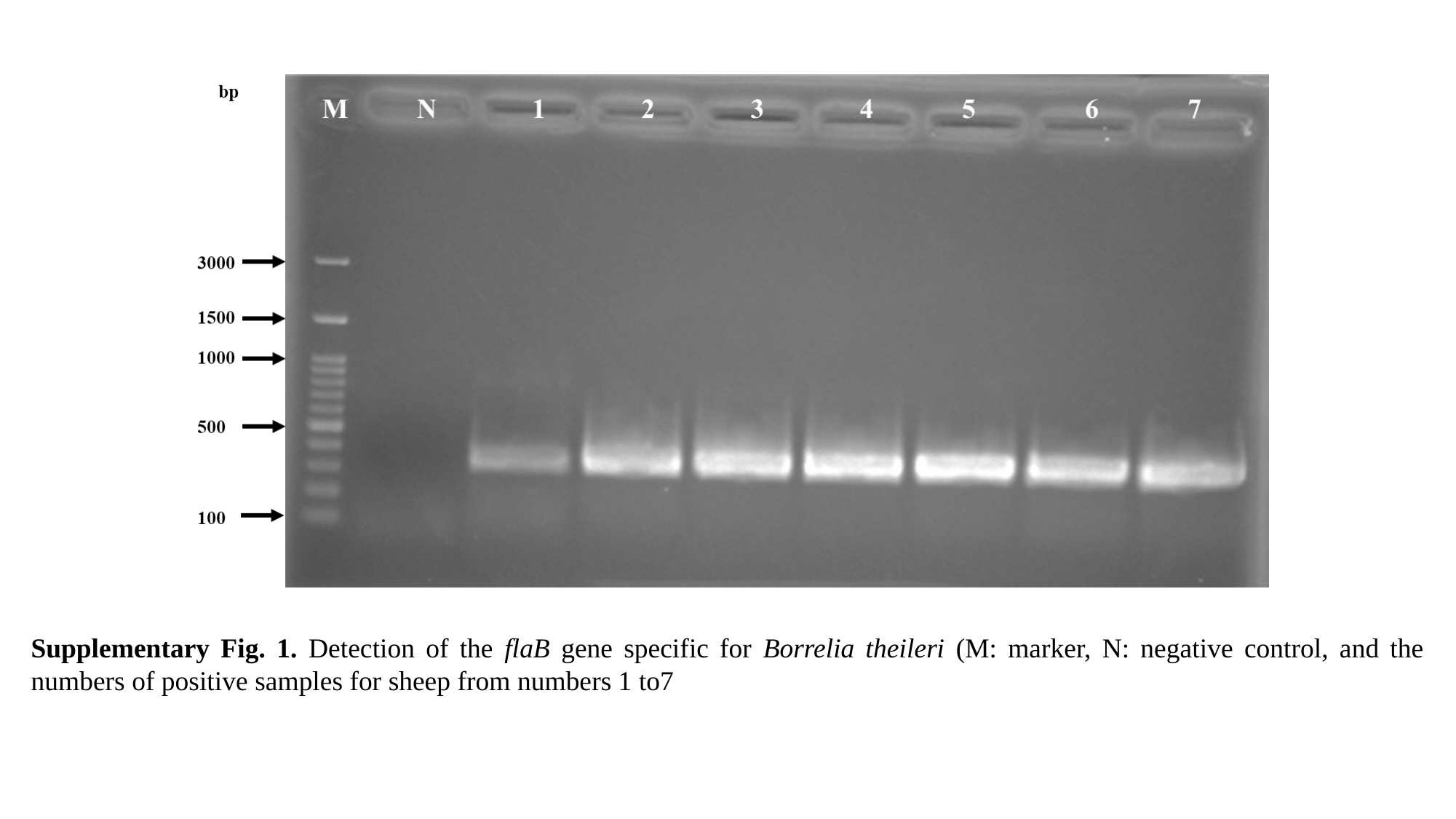

Supplementary Fig. 1. Detection of the flaB gene specific for Borrelia theileri (M: marker, N: negative control, and the numbers of positive samples for sheep from numbers 1 to7

## Slide 2
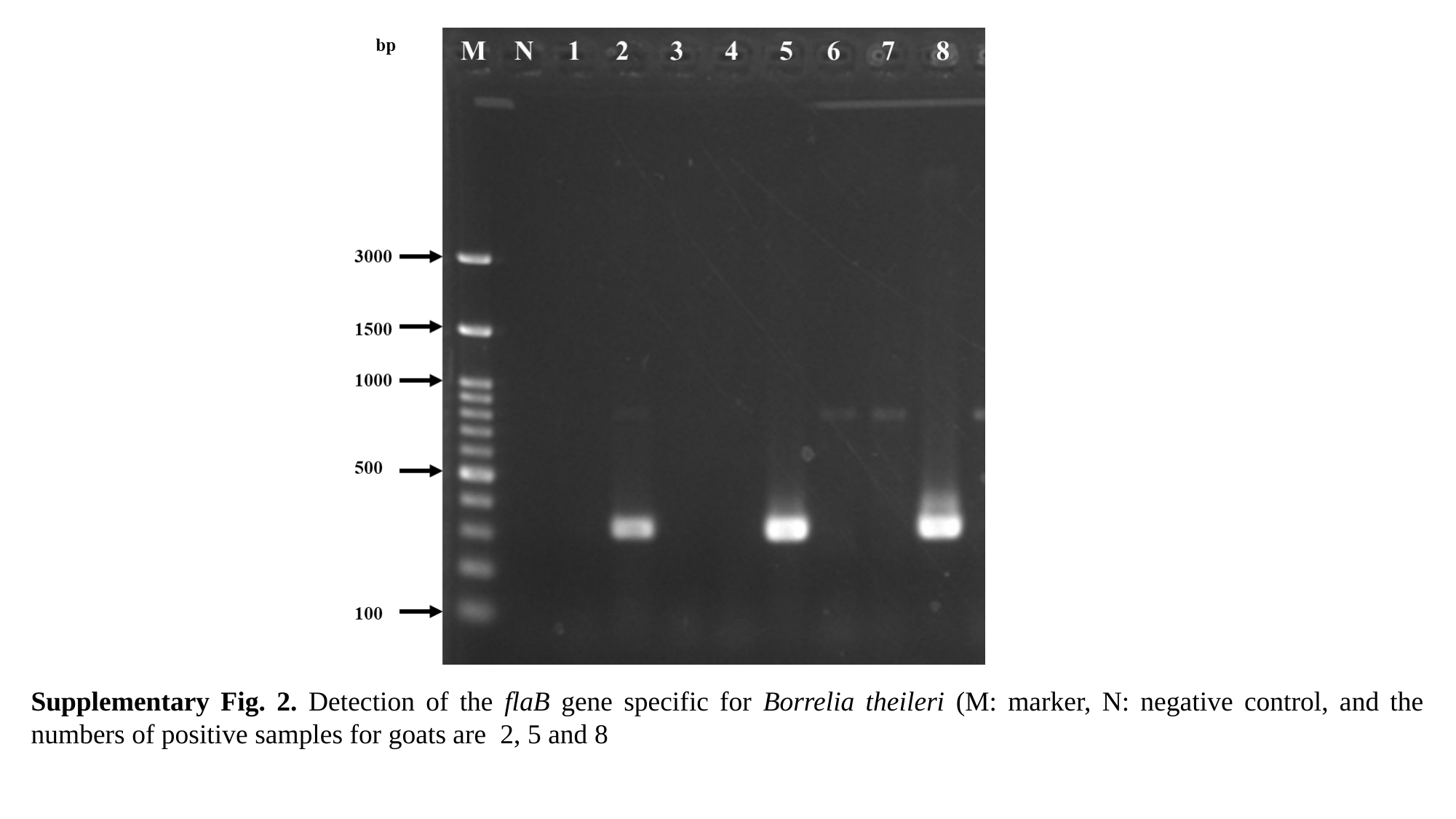

Supplementary Fig. 2. Detection of the flaB gene specific for Borrelia theileri (M: marker, N: negative control, and the numbers of positive samples for goats are 2, 5 and 8

## Slide 3
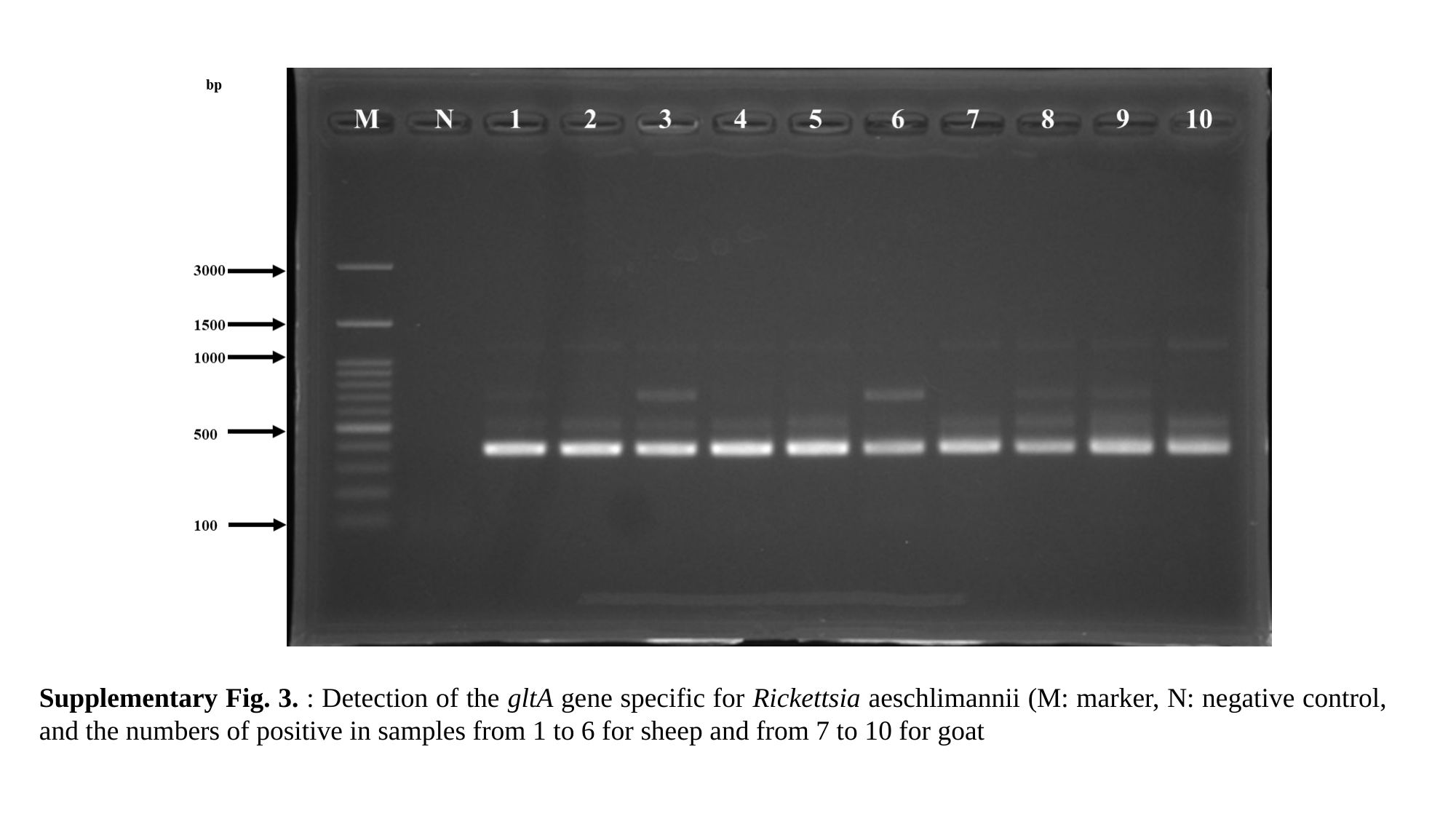

Supplementary Fig. 3. : Detection of the gltA gene specific for Rickettsia aeschlimannii (M: marker, N: negative control, and the numbers of positive in samples from 1 to 6 for sheep and from 7 to 10 for goat

## Slide 4
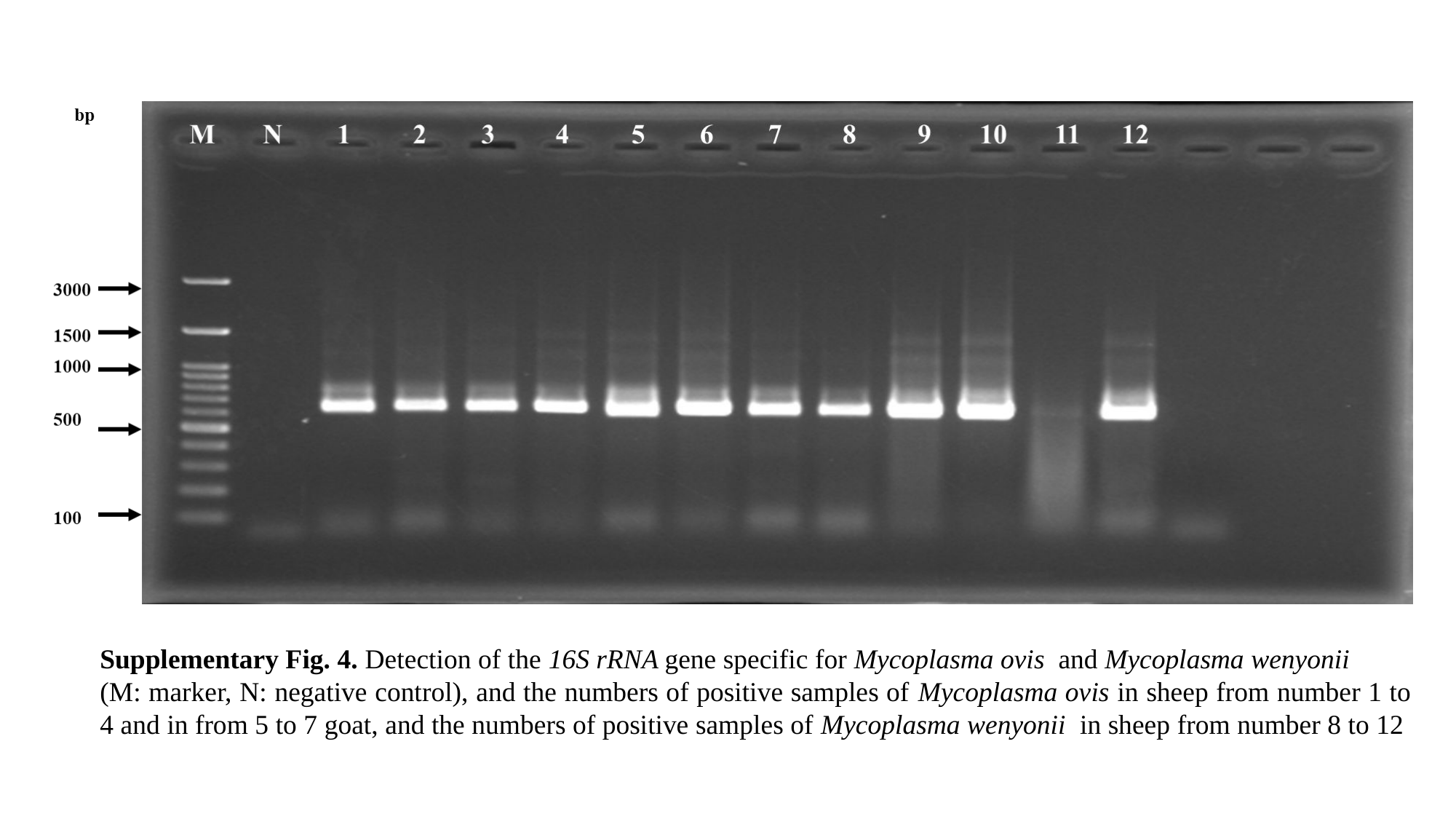

Supplementary Fig. 4. Detection of the 16S rRNA gene specific for Mycoplasma ovis and Mycoplasma wenyonii
(M: marker, N: negative control), and the numbers of positive samples of Mycoplasma ovis in sheep from number 1 to 4 and in from 5 to 7 goat, and the numbers of positive samples of Mycoplasma wenyonii in sheep from number 8 to 12
